# Is It Time to Beta Block the Septic Patient?

**DOI:** 10.1155/2015/424308

**Published:** 2015-10-18

**Authors:** Philip Pemberton, Tonny Veenith, Catherine Snelson, Tony Whitehouse

**Affiliations:** ^1^Department of Anaesthesia and Critical Care Medicine, University Hospitals Birmingham NHS Foundation Trust, Queen Elizabeth Hospital Birmingham, Mindelsohn Way, Birmingham B15 2GW, UK; ^2^Department of Infection and Inflammation, University of Birmingham, Edgbaston, Birmingham B15 2TT, UK; ^3^Department of Medicine, University of Cambridge, The Old Schools, Trinity Lane, Cambridge CB2 1TN, UK; ^4^College of Medical and Dental Sciences, University of Birmingham, Edgbaston, Birmingham B15 2TT, UK

## Abstract

Beta blockers are some of the most studied drugs in the pharmacopoeia. They are already widely used in medicine for treating hypertension, chronic heart failure, tachyarrhythmias, and tremor. Whilst their use in the immediate perioperative patient has been questioned, the use of esmolol in the patients with established septic shock has been recently reported to have favourable outcomes. In this paper, we review the role of the adrenergic system in sepsis and the evidence for the use of beta stimulation and beta blockers from animal models to critically ill patients.

## 1. Introduction

Although the mortality from septic shock has fallen in recent years, this has been through improved detection and earlier antibiotic therapy. Despite intensive research over 20 years, interventions to alter the course of sepsis once the immune response has initiated have not been found. Recently, a single centre phase-II study from Italy [1] reported that beta-adrenergic blockade in patients with septic shock who continued to have elevated heart rates after standard fluid resuscitation caused improvements in cardiac performance, plasma lactate clearance, and a reduction in vasopressor dependence, with no reported adverse effects. The study with 77 patients in each group was not powered to examine survival but nevertheless showed substantial reduction in short-term mortality (adjusted hazard ratio, 0.39; 95% CI, 0.26 to 0.59; *P* < 0.001; twenty-eight-day mortality was 49.4% in the esmolol group compared with 80.5% in the control group). This raises the question whether beta blockers could offer a new way of managing the critically ill patient with septic shock and if so how its benefits may arise.

In addition to the adrenoceptors found throughout the cardiovascular system, the adrenergic system is also a powerful modulator of the immune system [2]. Lymphoid organs (spleen, thymus, lymph nodes, and bone marrow) are predominantly innervated by the sympathetic system. With the exception of T helper type 2 (Th2) cells, the majority of lymphoid cells express beta-adrenergic receptors on their surface. The adrenergic system also modulates or regulates cell death, mitochondrial function, and inflammatory signalling [3]. Bone marrow production and differentiation of monocytes is influenced by the adrenergic system [4, 5] and immune cell apoptosis is at least partly mediated by catecholamines, via alpha-adrenergic and beta-adrenergic pathways. Although there has been a great deal of focus on the cardiovascular benefits of beta blockade in sepsis [6], the ubiquitous nature of the adrenergic system brings into question whether there are other mechanisms through which beta blockers may exert their influence.

## 2. The (Patho)physiology of the Sympathetic System during Septic Shock

Interaction between sympathetic nervous and immune systems is mediated with an effector arm consisting of catecholamines and inflammatory cytokines. In response to invading pathogens, there is up-regulation of sympathetic activity enabling the host to mount a rapid and effective response. It contributes to the early clinical presentation in sepsis of flushing, and tachycardia and hypotension caused, in part, by sympathetic mediated vasodilatation. That is to say that the cross-talk between the sympathetic system and the immune system is “physiological” rather than “pathological” in the first instance. However, there comes a point at which such an effector system begins to cause injury to the host [7]. The continued elevation of catecholamines observed in some septic patients becomes unfavourable and for some reason does not down regulate [8].

The pathophysiology of septic shock includes excessive sympathetic outflow and high concentrations of plasma catecholamines leading to vasodilatation followed by vasoconstriction, vascular hyporeactivity, myocardial depression, and autonomic dysfunction [7, 8]. The recommended treatment for fluid-unresponsive sepsis-related hypotension is norepinephrine [9], but this agent has numerous adverse effects including direct myocardial damage, insulin resistance, thrombogenicity, immunosuppression, and enhanced bacterial growth [10]. This hypercatecholamine state is in part also responsible for numerous compensatory metabolic alterations characteristic of the stress condition, including the dysregulation of glycaemic control seen after injury and sepsis [11, 12]. Some of these adverse effects could be attenuated by beta-adrenergic blockade, since this enables heart rate control [13] and limits adverse events related to sympathetic overstimulation [10].

It was noted many years ago that epinephrine enhanced bacterial infections [14] and decreased the number of bacteria necessary for a lethal dose in both* Clostridia* species and pathogenic aerobic organisms. Catecholamines have been demonstrated to enhance biofilm formation and stimulate bacterial growth in* Staphylococcus epidermidis* [15]. Growth of* Yersinia enterocolitica* [16] is enhanced by dopamine and norepinephrine (but not ephedrine), an effect mediated by removal of iron from lactoferrin and transferrin by the catechol moiety and its subsequent acquisition by bacteria [17].* Escherichia coli* O157:H7 and* Salmonella enterica *also have enhanced growth with catecholamines.

High plasma catecholamine levels have been noted in septic animals [18] and humans [8]. Boldt and colleagues observed elevated and persistently high plasma catecholamine levels in nonsurvivors in a critically ill patient population many of whom also received catecholamines as part of their treatment [19]. In septic shock patients, arterial norepinephrine levels are significantly associated with a greater mortality [8]. The extent and duration of catecholamine therapy and tachycardia are all independently associated with poor outcomes in critically ill patients [8, 20–22]. Concerns have been raised about the use of catecholamines in the treatment of septic shock [10].

## 3. Beta Blockers, Sepsis, and the Immune System: Nonhuman Studies

There has been longstanding interest in beta blockade and sepsis. As long ago as 1969, Berk et al. [23] used propranolol in a lipopolysaccharide (LPS) dog model and found that the beta blocker significantly improved survival when it was started 60 minutes after LPS insult. Propranolol also prevented hypotension and reduced fluid requirements. More recently, selective beta-1 blocking by esmolol decreased circulating TNF-alpha and IL-1beta concentrations in septic rats [24], reducing heart rate and blood pressure. Esmolol also protected LPS treated pigs from cardiovascular decline in a five hour model [25] so that in esmolol-treated animals the cardiac index had decreased by 9% after 3 hours and by only 2% at the end of the study; in controls the cardiac index had reduced by 14% and by 27%, respectively (*P* = 0.870). This was despite a decrease in heart rate of 20% in the esmolol group and an increase in heart rate of 22% in controls (*P* < 0.001). Continuous infusion of esmolol initiated after septic insult improved survival at 5 days in a murine model [26] and noted an increase in the NFkappa B pathway. Similarly, landiolol, another selective beta-1-blocker, also decreased circulating levels of TNF-alpha, IL-6, and high mobility group box 1 in a rat model of endotoxin-induced sepsis [27]. Pretreatment with atenolol or metoprolol did not alter survival in a cecal ligation and puncture (CLP) rat model [28] given 2 hrs before CPL but the median time to death was increased by 33 hrs in metoprolol-treated rats (*P* = 0.03). Metoprolol pretreatment reduced hepatic expression of proinflammatory cytokines and lowered plasma IL-6 (both *P* < 0.05). Myocardial protein expression of IL-18 and monocyte chemoattractant protein-1, key mediators of cardiac dysfunction in sepsis, were also reduced (*P* < 0.05). In another study, atenolol had an anti-inflammatory effect by increasing IL-10 but had no effect on TNF-alpha or L-6 concentrations in an ovine model with* E. coli* septicaemia [29].

Animal studies of the use of beta-agonists and beta blockade do not, however, give consistent and predictable results. Catecholamines have been demonstrated to* reduce* isolated human neutrophil function by* decreasing* free radical production, [30]. Early use of catecholamines at the same time as exposure of cells to LPS leads to the* downregulation* of synthesis of proinflammatory cytokines such as TNFalpha, IL-6, and IL-1 [31–33], and* upregulates* synthesis of anti-inflammatory cytokines (e.g., IL-10) [32, 33]. Schmitz et al. [34] demonstrated an* increased* mortality in mice treated with propranolol undergoing CLP when beta blocker treatment was started at the time of surgery. Authors [6] have speculated that initial fluid resuscitation was impaired and that the nonselective nature of propranolol reduced the cardioprotection proffered by beta blockers. This does not entirely explain all findings and ignores previous animal models [23] reporting benefit with propranolol.

Lang and colleagues [35] found that beta blockade exacerbated a sepsis-induced response in the presence of propranolol. They reported an* increase* in muscle IL-6 and TNF-alpha mRNA but did not alter the increment in IL-1alpha. Furthermore in another study [36], epinephrine infusion did not increase mortality at 48 hours in a CLP model in mice but induced alterations of splenocyte apoptosis, splenocyte proliferation, and IL-2 release. Subsequent treatment with propranolol* augmented* the epinephrine-induced increase of splenocyte apoptosis,* did not affect* the decrease of splenocyte proliferation and IL-2 release,* augmented* the release of IL-6, and antagonized the mobilization of natural killer cells. There was also a significant increase in mortality in propranolol treated animals.

## 4. Clinical Experience with Beta-Agonism

Studies emerged in the mid-1980s suggesting that patients treated with dobutamine and who could improve their cardiovascular performance had a better survival than those who did not [37, 38]. In particular, Shoemaker's often quoted paper [38] suggested that mortality from a protocol-guided resuscitation regimen could reduce mortality from 23% in the control group to 4% in surgical patients managed with a pulmonary artery catheter and the addition of dobutamine to achieve therapeutic goals that were supranormal values for cardiac output (>4.5 L/min·m^2^), DO2, (>600 mL/min·m^2^), and VO2 (>170 mL/min·m^2^). A subsequent larger resuscitation study [39] also demonstrated improvements in patients admitted through the emergency department and resuscitated according to a CVP based protocol. Rivers' paper [39] now forms the basis for fluid resuscitation in the Surviving Sepsis Campaign Guidelines for the management of severe sepsis and septic shock [9].

Most intensivists agree that the act of starting dobutamine is not the intervention that improves survival but it is the* ability* to achieve supernormal physiological status that selects out survivors. Shoemaker's study was followed shortly after by a study from the UK [40] which failed to demonstrate improved mortality in patients with established sepsis. Indeed, their results suggested that aggressive efforts to increase oxygen consumption could have been detrimental as the in-hospital mortality was lower in the control group (34 percent) than in the treatment group (54 percent) (*P* = 0.04; 95 percent confidence interval, 0.9 to 39.1 percent). One important difference, as shall be discussed later, is the timing of intervention. Hayes's paper [40] specifically intervened on patients who had been admitted to ICU and not those admitted from the emergency department.

There were a number of small studies towards the end of the last century that were summarised [41] as “Insufficient evidence exists to support goal-directed therapy with vasopressors and inotropes in the treatment of sepsis syndrome.” A later analysis by the Cochrane collaboration [42] concluded “Probably the choice of vasopressors in patients with shock does not influence the outcome….” In the days following Shoemaker's groundbreaking findings, it was therefore assumed that the use of beta agonism was appropriate for the management of sepsis despite the fact that Shoemaker studied patients in the perioperative period. There remain no well-conducted randomised trials comparing beta-agonism with placebo.

Although not directly studying sepsis, the Beta Agonist Lung injury Trial 2 (BALTI-2) [43] was a multicentre randomized controlled trial comparing the selective beta-2 agonist salbutamol with placebo (0.9% saline) in patients with Acute Respiratory Distress Syndrome (ARDS). Approximately 25% of the cases included in the trial had ARDS as a result of sepsis and almost 50% as a result of pneumonia. The study was stopped early (after recruitment of 326 of a planned 1334 patients) due to increased mortality in the treatment arm (10.9% (95% CI 1.0% to 20.4%) absolute increase (34.2% versus 23.3%) in 28-day mortality). 14.2% of patients in the salbutamol arm (versus 1.2 in the placebo) had the beta agonist stopped due to tachycardia (HR > 140 bpm) with 8.6% having treatment withdrawn due to arrhythmias.

A recent study has suggested that vasopressin used as a norepinephrine-sparing agent may improve outcomes from less severe septic shock. The overall conclusion of the Vasopressin versus Norepinephrine Infusion in Patients with Septic Shock (VASST) [44] study was that there was no mortality benefit by use of less norepinephrine. However, in the prospectively defined stratum of less severe septic shock, the mortality rate was lower in the vasopressin group than in the norepinephrine group at 28 days (26.5% versus 35.7%, *P* = 0.05); there continues to be a concern that sympathetic agonism is detrimental to at least some patients with septic shock.

## 5. What Is the Clinical Evidence for Beta Blockers in the Noncardiac Critically Ill Patient?

The normal myocardial expression of beta-1 and beta-2 adrenoceptors is in the approximate ratio of 3 to 1. In severe heart failure, this changes to 3 to 2 [45]. Sympathetic stimulation causes post synaptic noradrenergic beta-1 receptor stimulation whereas beta-2 adrenoceptors, located at extra junctional as well as post synaptic sites, are stimulated by circulating catecholamines [46]. This relative downregulation of beta-1 adrenoceptors may well be a protective mechanism designed to shield the heart from a high sympathetic drive. Iatrogenically mimicking this with the use of beta blockers may protect the heart from sympathetic overstimulation in septic patients. An elevated heart rate is associated with worse outcomes in septic patients [13]. As the severity of sepsis increases (SIRS to sepsis to septic shock), there is reduced heart rate variability and sympathetic activation worsens [47]. Excessive tachycardia seen in many septic patients reduces diastolic filling time, increases myocardial oxygen consumption, and may result in tachycardia induced cardiomyopathy [48].

Drugs that are antagonists to beta receptors have different affinities for beta-1 and beta-2 subtypes. Esmolol has a selectivity of beta-1 to beta-2 ratio of 33 and a half-life (*t*1/2) = 9.19 min [49] with landiolol having a greater affinity for the beta-1 receptor of beta-1 to beta-2 ratio 255 and *t*1/2 = 3.96 min [50]. In comparison, propranolol is 74–380 less selective for beta-1 when compared with landiolol and 39–263 when compared with esmolol [51]. Using recombinant cells expressing beta-1 and beta-2 receptors, Smith and Teitler [52] determined that bisoprolol was 19 times more selective for the beta-1 receptor and metoprolol 6 times more selective for the beta-1 receptor when compared with propranolol. It is hardly surprising then that in combination with the proportions of beta-1 to beta-2 receptors changing in health and disease, the response to beta blockade will vary depending upon how established sepsis is and which drug is selected.

The link between the adrenergic and immune systems requires further investigation: for example, beta blockade has also been shown to reduce proinflammatory cytokines in heart failure [53], critically ill trauma patients [54], and appears to have a beneficial effect on the T helper 1/T helper 2 ratio [55]. In a small trial including 55 critically injured patients at increased risk for heart disease, administration of metoprolol or esmolol decreased serum interleukin- (IL-) 6 levels [56]. A heart rate above 95 bpm is associated with major cardiac events in critically ill patients [22] but this is likely to be the result of effects on many pathways rather than solely improving myocardial oxygen utilisation.

Christensen and colleagues [57] performed a retrospective study in their ICU on 8087 patients over 6 years. In this case-matched study of 3112 patients (1556 users of beta blockers and 1556 nonusers), the 30-day mortality was 25.7% among beta blocker users and 31.4% among nonusers (OR 0.74 (95% CI: 0.63 to 0.87)). The OR was 0.69 (95% CI: 0.54 to 0.88) for surgical ICU patients and 0.71 (95% CI: 0.51 to 0.98) for medical ICU patients. The OR was 0.99 (95% CI: 0.67 to 1.47) among users of nonselective beta blockers, and 0.70 (95% CI: 0.58 to 0.83) among users of cardioselective beta blockers.

Herndon and colleagues successfully used propranolol to reduce heart rate by 20% in burned septic children and demonstrated attenuated hypermetabolism and reversal of muscle-protein catabolism both in the short-term [58] and over 12 months [59]. A retrospective analysis [60] examining 43 patients either already treated with (21 patients) or commencing beta blockers for other conditions in hospital (such as hypertension and tachyarrhythmias, 22 patients) suggested on multivariate analysis that pretreatment with beta blockers was associated with a significant decrease in fatal outcome and healing time.

Esmolol has undergone an evaluation of its safety in septic patients in several small studies; two from Europe [61, 62] and the authors understand that two case series have reported the safe use of esmolol in septic patients in China [63, 64]. In his follow-on study, Morelli [1] studied patients admitted to his hospital with septic shock who had received haemodynamic optimisation of fluid resuscitation and vasopressor administration to maintain a MAP ≥ 65 mmhg, CVP ≥ 8 cm H_2_0, pulmonary artery wedge pressure (PAWP) ≥ 12 mmHg, and mixed venous saturations (SvO2) > 65%. The primary outcome was reduction in heart rate within 24 hrs which was achieved. Surprisingly however, norepinephrine and resuscitative fluid volume requirements were also reduced (median area under the curve (AUC) for norepinephrine of −0.11 *μ*g/kg/min versus −0.01 *μ*g/kg/min, *P* = 0.003. Median AUC for fluid 3975 mL/24 hrs versus 4425 mL/24 hr, *P* < 0.001). Stroke volume, systemic vascular resistance, and left ventricular stroke work indices were all higher in the esmolol group whilst maintaining MAP ≥ 65 mmHg. In spite of a predictably reduced oxygen delivery (Do2) (median AUC −100 mL/min/m^2^ versus −32 mL/min/m^2^, *P* < 0.001), lactate, base excess, and arterial pH were all higher in the esmolol group. Although not powered to discover a mortality outcome, the authors also reported a substantial reduction in mortality with a twenty-eight-day mortality of 49.4% in the esmolol group and 80.5% in the control group.

## 6. Conclusion

Recent improvement in the mortality from sepsis has largely come from increased awareness, early management, and rationalisation of existing supportive measures. Despite extensive research, the literature is littered with numerous therapies that brought initial hopes of disease modulation only to be found not to make patients survive longer and, in some cases, increased mortality. Beta blockers have been shown to have immunomodulatory actions in addition to their cardiovascular effect and a phase II clinical trial [1] has suggested that beta blockers may have an important role in the treatment of the patient with septic shock. This study has raised questions and stimulated interest into why, when, and in whom beta blockers are beneficial. In particular, it raises the immediate question: (1) is the effect specifically and only seen with esmolol or would other beta-1 specific antagonists also prove beneficial? (2) When should beta blockers be started? (3) Does the persistence of tachycardia at 24 hours following resuscitation and presentation with septic shock select out a group of patients genetically predisposed to do worse in sepsis and benefit from beta blockade?

Morelli's findings are tantalising to ICU physicians, some of whom are already using beta blockade in patients with established sepsis. Whether their findings are borne out by larger, multicentre studies remains to be seen.
